# Socioeconomic inequalities in health outcomes among Thai older population in the era of Universal Health Coverage: trends and decomposition analysis

**DOI:** 10.1186/s12939-023-01952-0

**Published:** 2023-08-02

**Authors:** Sirinya Kaikeaw, Sureeporn Punpuing, Chalermpol Chamchan, Pramote Prasartkul

**Affiliations:** Institute for Population and Social Research, Mahidol University, Nakhon Pathom, Thailand

**Keywords:** Socioeconomic health inequality, UHC, Older persons, Decomposition of concentration index, Thailand

## Abstract

**Background:**

Thailand’s Universal Health Coverage (UHC) has been achieved since 2002 when the entire population are covered by three main public health security schemes: (1) Civil Servant Medical Benefit Scheme (CSMBS); (2) Social Security Scheme (SSS); and (3) Universal Coverage Scheme (UCS). Citizens have access to healthcare services at all life stages and are protected from catastrophic expenditure and medical impoverishment. However, there are health inequalities in both health outcomes and access to healthcare among older Thais. This study aims to: (1) assess the degrees of socioeconomic inequalities in health outcomes among the older Thai population during the period of Thailand’s UHC implementation (2003–2019), and (2) explain socioeconomic inequalities in health outcomes through decomposition of the contributions made by Thailand’s UHC policy and other health determinants.

**Methods:**

Data sets come from a four-year series of the National Health and Welfare Survey (HWS) between 2003 and 2019. The health outcome of interest was obtained from the Thai EQ-5D index. The Erreygers’ concentration index (CI) was used to calculate the socioeconomic inequality in health outcomes. Multivariate methods were employed to decompose inequalities.

**Results:**

Findings indicated Thai older adults (aged 50 and older) are healthier during the UHC implementation. Better health outcomes remain concentrated among the wealthier groups (pro-rich inequality). However, the degree of socioeconomic inequalities in health outcomes significantly declined by almost a factor-of-three (from CI = 0.061 in 2003 to CI = 0.024 in 2019) after the roll-out of the UHC. Decomposed results reported that Thailand’s UHC, urban residence, and household wealth were major contributors in explaining pro-rich inequalities in health outcomes among Thai older adults.

**Conclusions:**

Older persons in Thailand have better health while health inequalities between the rich and the poor have substantially decreased. However, there is inequalities in health outcomes within all three national health security schemes in Thailand. Minimizing differences between schemes continues to be a crucial cornerstone to tackling health inequalities among the older population. At the same time, making Thailand’s UHC sustainable is necessary through preparing financial sustainability and developing health resources to better serve an ageing society.

## Background

The persistence of inequalities in population health is currently a major challenge for public health worldwide. Especially, health inequalities among older persons are pronounced. In the context of reaching old age, inequalities are often reflected by the cumulative toll of lifelong socioeconomic disadvantages and vulnerability (e.g., poverty). This cumulative effect will manifest itself in a wide range of health problems, diseases, and longevity [1, 2]. The main causes of health inequalities are strongly associated with differences in socioeconomic status [3]. The best way to reduce health inequalities across a socioeconomic hierarchy is through an effective health policy as “Universal Health Coverage (UHC)” [4].

Thailand, like many other middle-income countries, has been experiencing a rapidly aging population, with more attention beginning to focus on the health of older persons. 19% of Thai people were aged 60 years and older in 2022, and it is projected to hit 20% in 2023, at which point Thailand becomes a “complete-aged society”[5]. More than half of the older population in Thailand suffers from chronic non-communicable diseases (NCDs); its prevalence is increasing from 51% in 2003 to 55% in 2019 [6, 7]. Incidences of chronic NCDs are leading causes of disability in older Thais [8]. What is more, the rapidly increasing older population is certain to bring with massive increases in the demand for more and different kinds of healthcare services as well as health expenditure in the future [9].

Concerning Thailand’s health system, the UHC policy was initially introduced in 2001, with coverage achieved in 2002. Its goal was to reduce health inequality and boost financial protection by providing access to essential healthcare for all citizens according to health needs regardless of ability to pay. Currently, almost all Thais are covered by one of three main public health security schemes: (1) Civil Servant Medical Benefit Scheme (CSMBS) for government employees (9%); (2) Social Security Scheme (SSS) for employees in the formal private sector (19%); and (3) Universal Coverage Scheme (UCS) for the rest of Thai citizens (71%). For older people, the majority of them are entitled to the UCS and enjoy a wide range of healthcare services [10].

Thailand’s UHC has successfully increased healthcare access through the implementation of the UCS. Population health has improved at large, as shown by the decline in overall mortality rates during 2001–2014 [11] and in the prevalence rates of the self-reported health limitations in older people (age 65 and over) [12], whilst substantially improving perceived well-being [13]. Despite universal coverage being achieved, some studies have indicated the persistence of socioeconomic inequalities in health-related outcomes as measured by self-reported health, specific reported diseases [14, 15], and self-reported oral health status [16]. Furthermore, these scholars found the highest health inequalities among those aged 60 years or older, particularly in women.

Thai older persons tend to be more vulnerable to health inequalities, they frequently face multiple barriers in accessing timely and adequate healthcare they need, particularly for those older people in rural areas with socioeconomic disadvantages and with mobility constraints [17, 18]. Although all older Thais – just as all Thai citizens – are covered by the UCS by default, health inequalities are also visible among the older persons themselves [17]. Furthermore, most health inequalities in old age are not random, but it is associated with the accumulation of socioeconomic disadvantages throughout a lifetime [1]. As age-related issues, the older persons are frequently more at risk of health problems and significantly need a variety of healthcare services [19]. Moreover, Thai older people had higher unmet healthcare needs than younger groups [20], and their healthcare utilization declined when reaching to the oldest old [17]. Together with age-related issues and cumulative socioeconomic disadvantages, this could contribute to more inequalities in a range of health outcomes in old age.

The existence of health inequalities in Thai older people is a challenge for the public health system, especially in providing adequate older healthcare needs regardless of their socioeconomic status [9]. Indeed, the Thailand’s UHC policy is partial to addressing health inequalities in term of healthcare access and health outcomes [21]. Various socioeconomic determinants might take a leading role in contributing to the persistence of health inequalities [3]. To better understand the inequalities in health outcomes among Thai older persons, it is necessary to identify and quantify factors behind these inequalities. That can provide valuable information for policymakers to design and target effective strategies to reduce inequalities that adversely affect older persons towards Thailand’s UHC policy.

The purpose of this present study is twofold: (1) to assess the degrees of socioeconomic inequalities in health outcomes among the Thai older population during during the period of Thailand’s UHC implementation (2003–2019), and (2) to explain socioeconomic inequalities in health outcomes through decomposition of the contributions made by Thailand’s UHC policy and other health determinants in 2003 and 2019.

### Development of Thailand’s Universal Health Coverage

Initially, there were four public health security coverage schemes in Thailand: the Medical Welfare Scheme (MWS), initiated in 1975, commenced with free medical care for the poor, and later extended to older population (age 60 years or older) in 1992. Another scheme is the Civil Servant Medical Benefit Scheme (CSMBS) for government employees, retirees, and their family members, established in 1978. Announced in 1983, the subsidized Voluntary Health Card Scheme (VHC) covers informal workers; and the Social Security Scheme (SSS), launched in 1990, covers employees in formal private sector [21]. Taken together, all four schemes should cover all Thai citizens. Nevertheless, at the end of 2000, nearly one in three Thai people, or around 18 million, were still uninsured and not enrolled in one of these schemes [21, 22]. Filling gaps in subsidized health coverage, Thailand introduced the UHC policy for the entire population as part the health system reform in 2001 (i.e., the Universal Coverage Scheme, or UCS). Finally, Thailand achieved the coverage in 2002 when the whole Thai citizens were under one of the three main public health security schemes throughout their life: the CSMBS, SSS, and UCS [23].

As the main public health security schemes in the country, the UCS was established to reduce health inequalities by offering equitable access to healthcare services for all Thai people when they need it without suffering financial hardship [21, 22]. The UCS is financed by an annual budget allocation from general tax revenue. The SSS is financed by compulsory tripartite contributions from employees, employers, and government, each at 1.5% of payroll (total of 4.5%). While, the CSMBS is fully funded from general tax revenue and pays providers based on a fee-for-service. Currently, the benefits packages of all schemes are comprehensive, covering curative services (such as outpatient (OP) and inpatient (IP) services, emergency services), high-cost treatments, and health promotion and disease prevention [24, 25]. Subsequently, there remain challenges in the different details between each scheme such as using different sources of finance, the benefit package, capital expenditure, etc. [26]. CSMBS members seem to have the most privilege from healthcare services with unrestricted access to public hospitals and almost unlimited provider choice [25]. Key characteristics of three public health security schemes are described in Table 1.

**Table 1:** Thailand: Characteristics across three public health security schemes, 2020

Characteristics	CSMBS (1980)		SSS (1990)		UCS (2002)
**Scheme nature**	Fringe benefit		Compulsory contribution		Social welfare
**Target group**	9%, government employee, pensioners, dependents		19%, formal workers (Article 33) and informal workers (Article 39), excluding dependents		71%, people who are not covered by CSMBS and SSS
**Financing sources**	General tax revenue		Tripartite, 4.5% payroll, 1.5% each		General tax revenue
**Expenditure per capita** ^**(1)**^	12,676 Baht (US$ 384) (in 2017)		3,355 Baht (US$ 102) (in 2017)		3,600 Baht (US$ 109) (in 2020) ^(2)^
**Providers**	Public and private providers		Competing public, private hospitals (60% in the private sector)		Mostly (94%) public network, typical District Health System (DHS) (district hospitals and health centers)
**Provider payment**	OP: Fee-for-service IP: diagnostic-related groups (DRG) with multiple cost bands		OP: Capitation IP: DRG within global budget		OP and health promotion and prevention: Capitation (age adjusted) IP: DRG within global budget Fee schedule for specific high-cost procedures
**Benefit package**	Comprehensive with no explicit exclusion list, private bed covered		Comprehensive with a small exclusion list: OP, IP, accident and emergency, high-cost care		Comprehensive with a small exclusion list: similar to SSS; including clinical prevention and health promotion (Note: health promotion and prevention cover all Thai population in every scheme)

Noted: ^(1)^ 1 US$ = 33 Baht; ^(2)^ The health promotion and prevention services per capita budget (453 Baht or US$ 14) is for all Thai (not only UCS). Article 33 covers those who are non-government workers in the formal sector; Article 39 covers those who are informal workers and previously worked under Article 33, and wish to continue to submit a contribution for being an insured person

Sources: adapted from [10, 21]

The achievement of Thailand’s UHC is remarkable, in terms of the coverage, benefits package, healthcare utilization, and financial risk protection [27]. The UCS has been found to have a positive impact on healthcare utilization. Utilization among people under the UCS increased from 71.5% to 2003 to 81.2% in 2019 [28]. Moreover, UCS utilization was largest for the older population [29], and use of primary healthcare services was greater among rural people and the urban poor [30]. Many households were protected from catastrophic health expenditures and impoverishment due to costs of medical care [22, 31, 32]. Nevertheless, other studies have reported that socioeconomic health inequalities in health-related outcomes and healthcare utilization persist, despite universal coverage being achieved [14–16, 33, 34].

## Methods

### Data source

Data sets to carry out the analysis were taken from a four-year series of national Health and Welfare Survey (HWS) between 2003 and 2019 which covered the full period of implementation of the UHC. The HWS has been initiated and conducted by the National Statistical Office of Thailand (NSO). These HWSs collected data from nationally representative more than 60,000 respondents (aged 15 or older) from over 19,000 households in each round of survey by employing two-stage stratified sampling. Since this study mainly focuses on older people, Thai people aged 50 years or older who responded to the self-assessed health information in the HWS made up the sample in this analysis. The sample consists of 12,450 respondents in 2003; 12,535 in 2006; 29,670 in 2015^1^; and 16,324 in 2019. Data from samples were weighted to represent the country’s population using sampling weight variable provided with the HWS.

### Measurement of health outcome: Thai EQ-5D index

The EuroQol-5 Dimensions (EQ-5D) was employed to measure health outcomes of older adults. It is a commonly used multidimensional health questionnaire, which considers five dimensions including (1) mobility, (2) self-care, (3) usual activities, (4) pain/discomfort, and (5) anxiety/depression. Each of these domains has five response options: no problem (Level 1), slight problem, moderate problem, severe problem, and extreme problem (Level 5), typically called “EQ-5D-5L”. Responses from EQ-5D-5L are coded as a 5-digit code (combined from all 5 dimensions) expressing health states. There are 3,125 health states (5^5^). The value of the best health state is “11111”, while the worst health state is “55555”. However, these health states need to be converted into a single summary index, called “EQ-5D index.” This index score based on the preferences of the general population of a country [35, 36].

As employing four rounds of HWS, the EQ-5D-5L instruments have been provided in surveys. It was employed to measure health outcomes in this study. All five dimensions of the EQ-5D-5L instruments have been evaluated the internal consistency [37] with high reliabilities^2^ in four rounds. Responses from EQ-5D-5L instruments provided a total of 3,125 health states (with a 5-digit code) that were converted into the Thai preference-based value sets, developed by Pattanaphesaj [38]. Thai EQ-5D index scores in all four rounds have a score ranging from − 0.283 (the worst health) to 1 (the full health or the best health). A respondent having a higher index score is healthier than others. Therefore, the health outcome variable employed in this study is an indicator of ***better health***.

### Measurement of socioeconomic status (SES): household wealth index

Socioeconomic status was measured using a household wealth index, derived from housing characteristics (the materials of construction), housing facilities/infrastructure (sources of drinking water), and household ownership of durable goods. A Principal Component Analysis (PCA) was applied to compute the household wealth index in each year of HWS datasets. As PCA results, the household wealth index from the first principal component was transformed into quintiles generating. The first quintile represented the poorest group and the fifth quintile was the richest group [39].

### Potential deterministic variables

Social determinants of health (SDH), which is proposed by the World Health Organization (WHO), revealed factors that influence positively or negatively on health and health inequalities among different population groups [3]. In this study, SDH has been adopted to potential deterministic variables which are considered for explaining socioeconomic inequalities in health outcomes among older population. Thailand’s health security schemes were identified as a structural determinant of health accessibility in this study. It refers to three main types of public health security schemes in Thailand: Universal Health Coverage Scheme (UCS), Civil Servant Medical Benefit Scheme (CSMBS), and Social Security Scheme (SSS). As employing Health and Welfare Survey (HWS), the respondents were asked about the current health security scheme holding. Those who are not covered by CSMBS and SSS (Articles 33, and 39), as reporting unknown or holding private health insurance^3^, were included in the UCS group because all Thai citizens are eligible for the UCS by the National Health Security Act B.E. 2545 (A.D. 2002).

Other health determinants included respondents’ report on their demographic (age-sex), socioeconomic (household wealth, and level of education), geographic (urban-rural residence, regions) characteristics, and health conditions (having non-communicable diseases (NCDs)).

### Statistical analysis

In the recent health economic literature, several indices have been proposed to quantify the degree of health inequalities. The concentration curve (CC) and index (CI) are the most distinctive indices to measure the extent to which the health outcome is associated with inequality in a measure of socioeconomic status, typically called *the socioeconomic inequality in health* [40]. The CC was used to depict inequality by plotting the cumulative percent of the health (or other) variable of interest on its y-axis against the cumulative percent of the population, ranked from the poorest household wealth, and ending with the wealthiest on its x-axis. The CC forming a 45-degree-angle line represents perfect equality of health variable across people group. If the curve lies above the equality line, the inequality of the interested health variable is concentrated among the poor, or so-called “pro-poor” inequality, and the reverse is true or “pro-rich” while it lies below line of equality [41].

In addition, the degree of socioeconomic inequality in health outcomes can be summarized by the CI which is defined as twice the area between the CC and the 45-degree-angle line. This index has a potential value ranging from − 1.0 to 1.0, and a value of zero represents the equality. A negative (or positive) value indicates that the health outcomes variable of interest is concentrated among the worse-off population (the better-off) [40, 41].

This study employed the Erreygers’ concentration curve and index (2009) to measure the extent of socioeconomic inequality in health outcomes among older adults. Both Erreygers’ CC and CI are the most appropriate because they are more compatible with a bounded health variable as Thai EQ-5D index scores. Moreover, they considered under the condition that the scale of health variable has a finite lower and/or a finite upper limit [42, 43]. The formula of CI can be expressed as follows:

1 $$\text{C}\text{I}= \frac{8}{{n}^{2}\left(h\text{max }-h\text{min}\right)} \sum _{i=1}^{n}{h}_{i},{r}_{i}$$

Where *h*_*i*_ is the *EQ-5D index scores* of the *i*^*th*^ individual, $r$_*i*_ is the *i*^*th*^ individual’s rank in terms of *household wealth index. n* is number of persons.$h\text{max}$ and $h\text{min}$ are the upper limit of *EQ-5D index* (which is 1) and the lower limit of *EQ-5D index* (which is -0.283), respectively [42, 44]. It is important to highlight that results of CI for health outcomes could be expressed as the sum of contribution of various determinants by using decomposition approach.

The results of CI can be subsequently decomposed into various determinants that contribute to the socioeconomic inequality in health outcomes. The *decomposition method* was performed to quantify how each determinant contributes to the overall inequality [45]. Decomposing the CI into its contributing determinants commonly based on a regression model [41]. Given the fact that the health outcomes variable in this study is a bounded variable, the decomposition analysis is based on *Tobit regression model.* This model allows for the lowest and highest value of the EQ-5D index, so that estimates are not over the EQ-5D range. Moreover, the model acknowledged the presence of the ceiling effect^4^ that is a great deal of respondents had full health [46–48].

Afterword, the decomposition of CI for EQ-5D index scores was calculated by applying the Erreygers & Kessels’ formula as follow [49]:

2 $$ECI \left(y\right)=4{\sum }_{k}{\beta }_{k}^{m}{\stackrel{-}{x}}_{k}{C}_{k }+4G{C}_{\epsilon }$$

When ${\beta }_{k}^{m}$ is the marginal effects of the explanatory variables, ${\stackrel{-}{x}}_{k}$ is the mean of determinant ${x}_{ki}$, and ${C}_{k }$ is the mean of the concentration index for ${x}_{k}$. The explained component gives further information about the impact of each determinant on health outcomes (measured by its elasticity, ${\eta =\beta }_{k}^{m}{\stackrel{-}{x}}_{k}$), and the degree of unequal distribution of each determinant across socioeconomic status (measured by its CI, ${C}_{k }$). For the unexplained component, it can be expressed by $G{C}_{\epsilon }$ which is the generalized CI for the error term [49].

The results from formula **(2)** show the contribution of each determinant to the socioeconomic inequality in health outcomes. The contribution could be positive or negative as reflected in the sign of the CI and the association between determinants and health outcomes. In addition, even if the contribution of a determinant is large, but it has no impact on health outcomes or it is equally distributed between the rich and poor, this determinant will not be a key contributor in explaining overall socioeconomic inequality in health outcomes [14].

All analyses were conducted by using STATA 15.1 and Microsoft Excel. Also, sample weights were performed to make the results more representative of the country’s population.

## Results

### Characteristics of the study population

Table 2 presents weighted descriptive statistics of the basic characteristics of older adults (aged 50 and over) in 2003–2019. Most older adults (80.4 – 83.6%) were covered by the UCS which is eligible for all Thai citizens. About one-sixth of older adults (12.5–17.1%) were under the CSMBS, whereas only 1.9 – 6.4% were members of the SSS.

**Table 2:** Percentage distribute and mean of EQ-5D index (SD) of older adults by demographic, socioeconomic, geographic, and health characteristics in 2003–2019

Characteristics	2003 (n = 12,450)			2006 (n = 12,535)			2015 (n = 29,670)			2019 (n = 16,292)	
	%	Mean of EQ-5D index (SD)		%	Mean of EQ-5D index (SD)		%	Mean of EQ-5D index (SD)		%	Mean of EQ-5D index (SD)
Overall	100.0	0.832 (0.228)		100.0	0.867 (0.183)		100.0	0.934 (0.129)		100.0	0.941 (0.115)
***Healthcare accessibility***											
**Health security schemes**											
UCS	83.6	0.827 (0.229)		80.4	0.860 (0.188)		80.8	0.931 (0.132)		81.2	0.937 (0.118)
CSMBS	14.4	0.850 (0.229)		17.1	0.892 (0.160)		14.1	0.939 (0.121)		12.5	0.947 (0.109)
SSS	1.9	0.919 (0.152)		2.5	0.930 (0.115)		5.1	0.962 (0.078)		6.4	0.970 (0.076)
***Demographic and socioeconomic characteristics***											
**Sex-age interaction**											
Males age 50–59	22.2	0.907 (0.169)		20.8	0.918 (0.155)		18.7	0.963 (0.096)		18.6	0.971 (0.080)
Males age 60–69	13.3	0.847 (0.213)		13.4	0.886 (0.171)		13.0	0.942 (0.118)		13.8	0.950 (0.105)
Males age 70–79	6.8	0.770 (0.261)		6.6	0.813 (0.206)		6.9	0.901 (0.160)		6.2	0.910 (0.118)
Males age 80+	2.3	0.666 (0.348)		1.5	0.728 (0.257)		2.3	0.882 (0.167)		2.6	0.865 (0.173)
Females age 50–59	26.7	0.874 (0.184)		28.7	0.900 (0.144)		27.7	0.958 (0.090)		26.4	0.966 (0.081)
Females age 60–69	16.3	0.795 (0.240)		17.7	0.843 (0.184)		18.4	0.929 (0.121)		19.7	0.934 (0.119)
Females age 70–79	9.0	0.734 (0.253)		9.3	0.770 (0.224)		9.7	0.877 (0.175)		9.2	0.888 (0.148)
Females age 80+	3.4	0.630 (0.301)		2.1	0.706 (0.234)		3.2	0.820 (0.217)		3.5	0.833 (0.175)
**Mean of age (year)**	**61.8**			**61.4**			**62.2**			**62.3**	
**Household Wealth**											
Q1 (poorest)	25.4	0.790 (0.256)		31.2	0.823 (0.210)		22.6	0.910 (0.157)		20.0	0.919 (0.136)
Q2	27.4	0.821 (0.238)		23.3	0.875 (0.167)		21.0	0.928 (0.137)		20.7	0.934 (0.124)
Q3	17.1	0.830 (0.224)		16.7	0.877 (0.179)		16.4	0.940 (0.112)		18.9	0.949 (0.103)
Q4	15.4	0.867 (0.192)		14.3	0.891 (0.159)		19.1	0.940 (0.121)		19.6	0.943 (0.112)
Q5 (wealthiest)	14.6	0.893 (0.178)		14.6	0.914 (0.145)		20.9	0.954 (0.097)		20.8	0.959 (0.09)
**Education**											
No education	12.8	0.746 (0.276)		10.9	0.818 (0.219)		6.5	0.891 (0.183)		5.5	0.905 (0.147)
Pre and Primary level	77.5	0.836 (0.222)		76.4	0.865 (0.181)		74.6	0.931 (0.130)		72.7	0.936 (0.118)
Secondary level	5.7	0.899 (0.188)		8.5	0.919 (0.155)		10.5	0.954 (0.104)		12.5	0.962 (0.092)
Post-secondary	4.0	0.930 (0.133)		4.2	0.941 (0.102)		8.4	0.966 (0.077)		9.3	0.969 (0.080)
***Geographic characteristics***											
**Urban-rural residence**											
Urban	30.4	0.856 (0.216)		27.3	0.889 (0.170)		40.5	0.940 (0.122)		40.6	0.947 (0.107)
Rural	69.6	0.822 (0.233)		72.7	0.859 (0.187)		59.5	0.929 (0.133)		59.4	0.936 (0.120)
**Region**											
Bangkok	10.9	0.865 (0.216)		8.2	0.918 (0.146)		9.3	0.947 (0.107)		9.3	0.953 (0.091)
Central	23.1	0.852 (0.200)		23.4	0.874 (0.166)		25.3	0.927 (0.131)		26.0	0.939 (0.115)
North	21.0	0.806 (0.251)		22.5	0.869 (0.195)		22.4	0.945 (0.117)		21.5	0.942 (0.116)
Northeast	34.0	0.823 (0.233)		33.6	0.848 (0.196)		31.3	0.926 (0.142)		31.2	0.937 (0.116)
South	11.0	0.838 (0.226)		12.3	0.869 (0.167)		11.7	0.937 (0.120)		12.0	0.939 (0.125)
***Health conditions***											
**NCDs morbidity**											
No morbidity	56.2	0.900 (0.162)		56.9	0.910 (0.138)		54.5	0.965 (0.080)		56.7	0.968 (0.071)
Single morbidity	33.7	0.766 (0.258)		35	0.822 (0.207)		27.7	0.909 (0.146)		24.5	0.919 (0.135)
Multimorbidity	10.1	0.677 (0.291)		8.1	0.762 (0.244)		17.8	0.875 (0.182)		18.9	0.885 (0.158)

Noted: calculated from weighted samples

For the details of older adults’ characteristics, the proportions of older females were higher than males. Older adults were between 61.4 and 62.3 years on average. Approximately half of them were in both male and female aged 50–59 years. It is evident that over one-fourth of older adults in 2003 and 2006 tended to live in the poorest households, while about one-fifth of them found in 2015 and 2019. Most older adults had completed pre-and primary education. Furthermore, most older adults resided in rural areas, with one-third living in the Northeast region. More than two-fifth reported at least one NCDs morbidity. The proportions of reporting a single morbidity declined, while reporting multimorbidity grew by twofold.

### Trends in health outcomes of Thai older adults

Table 2 also shows the overall mean EQ-5D index scores with a conversion based on Thai preferences during 2003–2019. The EQ-5D index ranged from − 0.283 (the worst health) to 1 (full health). On average, the overall mean EQ-5D index scores increased from 0.832 to 2003 to 0.867, 0.934, and 0.941 in 2006, 2015, and 2019, respectively. It indicated that Thai older adults were averagely considered to live with better health outcomes during the implementation of UHC policy.

When focusing on the mean EQ-5D index scores in the light of public health security schemes, the mean EQ-5D index scores in each scheme increased over time. Moreover, the SSS members were found to have the highest mean score when compared to the CSMBS and UCS members. Even though the UCS members have the lowest health outcomes, the differences in mean EQ-5D index scores between the CSMBS and the UCS narrowed over the study period.

Older males had better health outcomes compared to females, and the mean EQ-5D index scores of both males and females reduced with age increase. Moreover, the wealthiest households and higher education tend to have better health outcomes. Living in an urban area and Bangkok commonly presented better health outcomes compared with other areas. Whereas, the lowest health outcomes appeared in the North (in 2003), and Northeast (in 2006–2019). As expected, older adults who have NCD conditions showed lower health outcomes, particularly those who are suffering from multimorbidity.

### Socioeconomic distribution of health security schemes

**Fig. 1:**
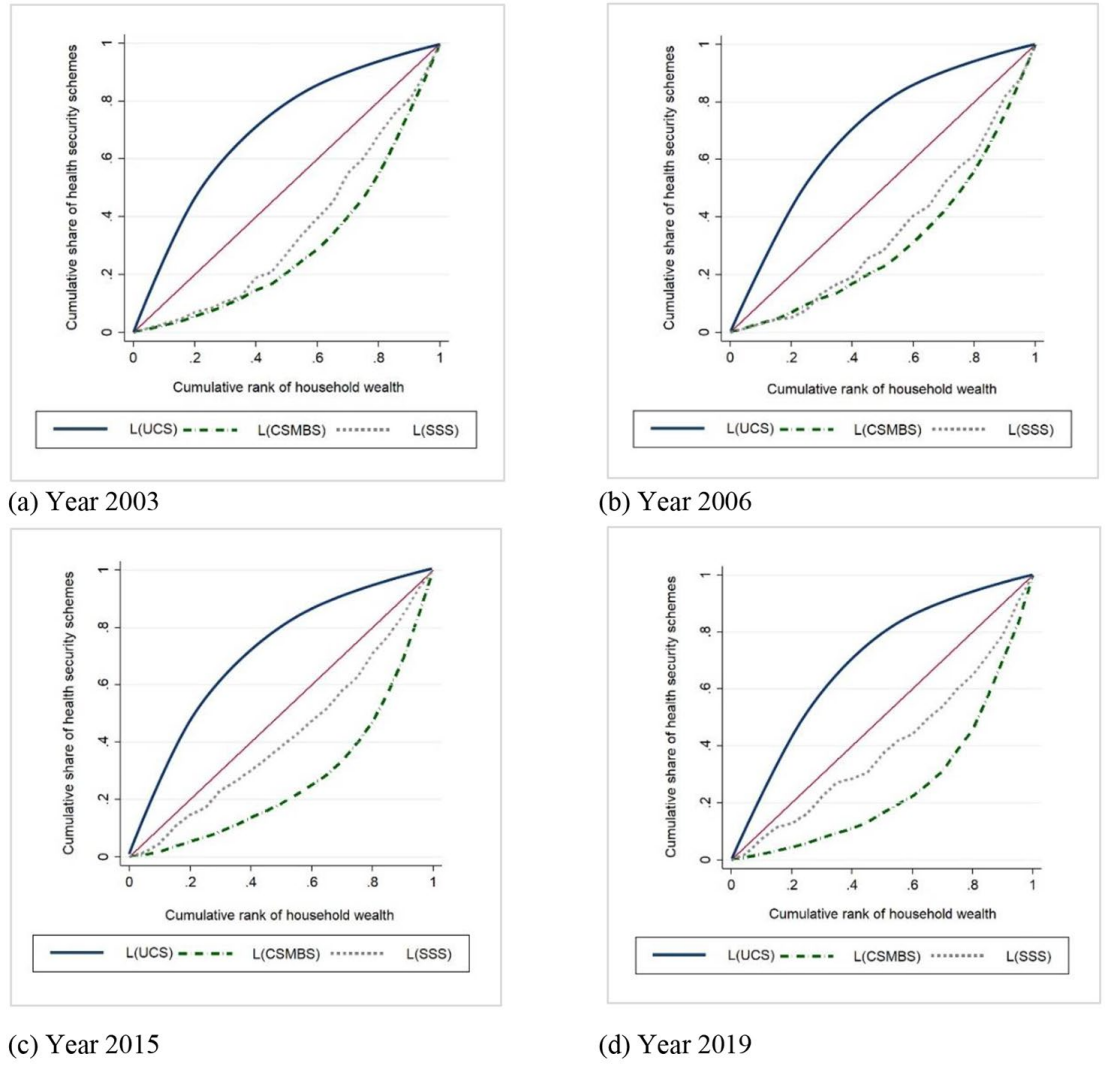
Concentration curves (CC) for health security schemes, 2003–2019

As illustrated in Fig. 1, we can see that the concentration curves (CC) of the UCS lied above the line of equality, whereas the CCs of the CSMBS and SSS were under the equality line. It indicated that older adults who are covered by the UCS were concentrated among the worse-off. In contrast, the CSMBS and SSS members belong to the better-off socioeconomically. It is interesting to note that these kinds of three health security schemes patterns appeared in all four periods of this study.

### Trend in the socioeconomic inequality in health outcomes in 2003–2019

**Fig. 2:**
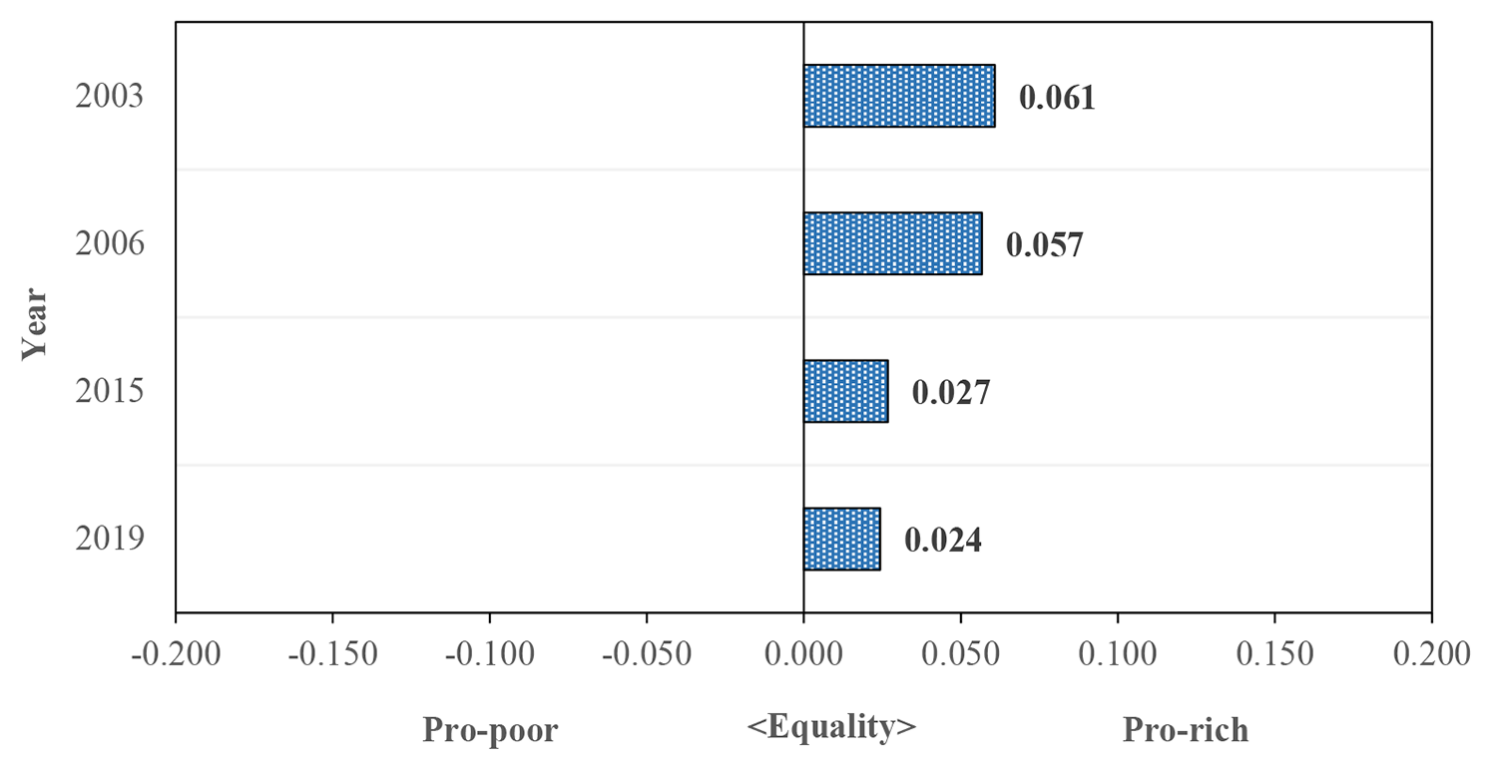
Erreygers’ concentration indices for health outcomes: 2003–2019

The results demonstrated that the concentration index (CIs) of overall health outcomes were significantly positive (pro-rich inequality) among older adults in Thailand. It concludes that having better health outcomes was concentrated among the richer groups. Moreover, the positive CIs of health outcomes decreased from 0.061 to 2003 to 0.057 in 2006, and then drastically dropped to 0.027 in 2011 and 0.024 in 2019 (Fig. 2). It is interesting to highlight that after the roll-out of the UHC in 2002, the degrees of socioeconomic inequality in health outcomes among older adults decreased and it is nearly equitable between the rich and the poor, especially in 2019. This states that older adults of both high and low SES in Thailand have almost an equity opportunity to be healthy.

### Decomposition of the socioeconomic inequality in health outcomes in 2003 and 2019

**Fig. 3:**
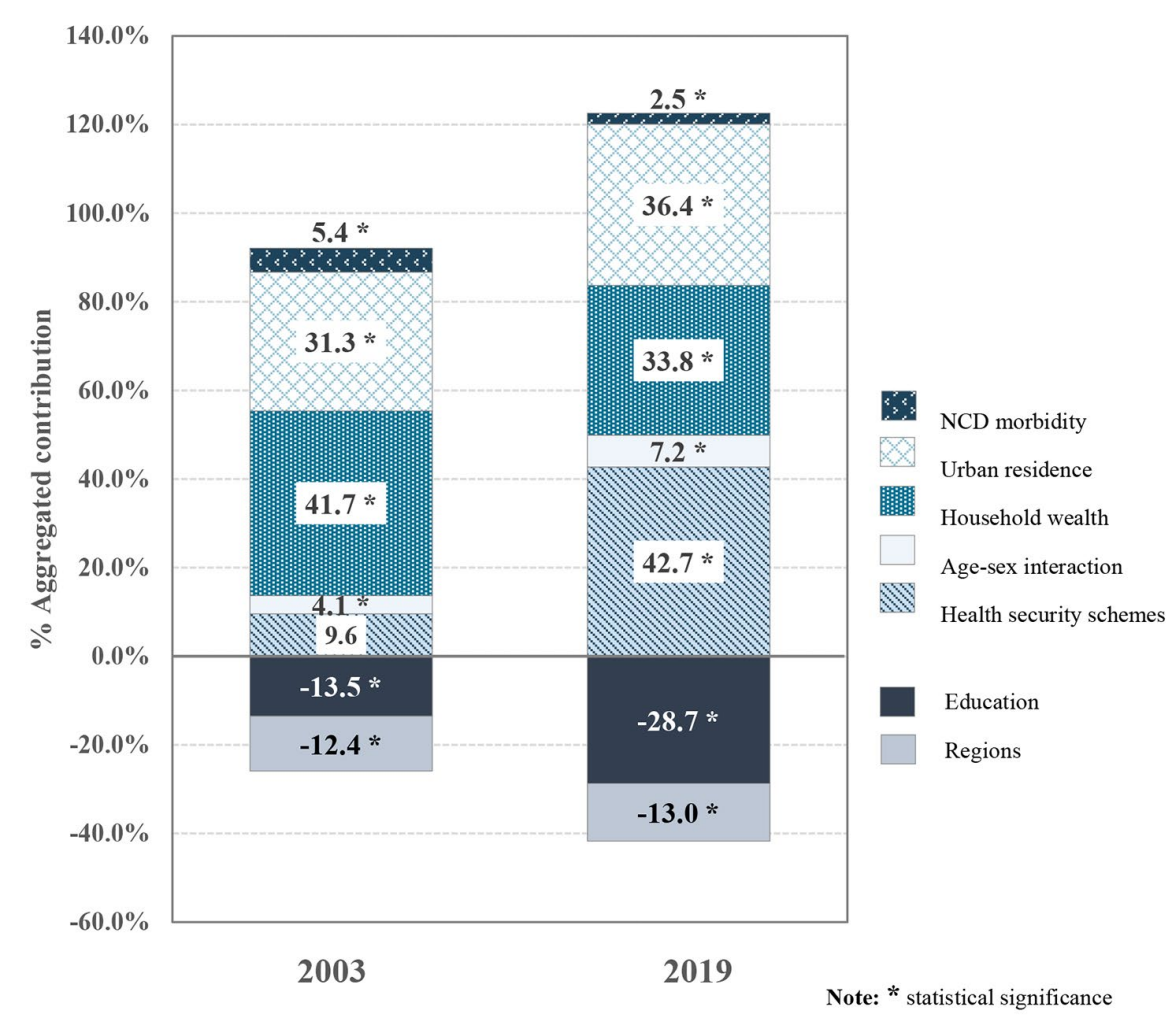
Percent contributions of each determinant to the socioeconomic inequality in health outcomes between 2003 and 2019

As mentioned above, the socioeconomic inequalities in health outcomes persist among older adults, although its degrees have lessened throughout the study period. Therefore, this study aims to find determinants derived behind the socioeconomic inequality in health outcomes in 2003 (CI = 0.061) and 2019 (CI = 0.024). The results of the decomposition of inequality in health outcomes was presented in Table 3 and Fig. 3.

**Table 3:** Decomposition of socioeconomic inequality for health outcomes in the Thai older population: 2003 and 2019

Determinants	2003							2019					
	**Marginal effect**(${\beta }_{k}^{m}$)		**Elasticity** $\left({\eta }_{k}\right)$	${C}_{k }$	**Con.**	**% Con.**		**Marginal effect**(${\beta }_{k}^{m}$)		**Elasticity** $\left({\eta }_{k}\right)$	${C}_{k }$	**Con.**	**% Con.**
**Healthcare accessibility**													
**Health security schemes**													
**CSMBS (ref.)**				0.246							0.236		
UCS	-0.006		-0.021	-0.273	0.006	9.5		-0.011	***	-0.035	-0.301	0.011	43.8
SSS	0.009		0.001	0.028	0.000	0.0		-0.016	***	-0.004	0.064	0.000	-1.1
	**Aggregated contribution**				**0.006**	**9.6%**		**Aggregated contribution**				**0.010**	**42.7%**
**Demographic characteristics**													
**Age-sex interaction**													
**Males age 50–59 (ref.)**				0.055							0.053		
Males age 60–69	-0.035	***	-0.019	-0.015	0.000	0.5		-0.019	***	-0.010	0.020	0.000	-0.9
Males age 70–79	-0.082	***	-0.022	-0.024	0.001	0.9		-0.045	***	-0.011	-0.034	0.000	1.6
Males age 80+	-0.169	***	-0.015	-0.005	0.000	0.1		-0.068	***	-0.007	-0.016	0.000	0.5
Females age 50–59	-0.017	***	-0.019	0.069	-0.001	-2.1		-0.003		-0.003	0.093	0.000	-1.1
Females age 60–69	-0.061	***	-0.040	-0.031	0.001	2.0		-0.023	***	-0.018	-0.016	0.000	1.2
Females age 70–79	-0.104	***	-0.037	-0.037	0.001	2.3		-0.052	***	-0.019	-0.055	0.001	4.3
Females age 80+	-0.168	***	-0.023	-0.011	0.000	0.4		-0.080	***	-0.011	-0.034	0.000	1.6
	**Aggregated contribution**				**0.003**	**4.1%**		**Aggregated contribution**				**0.002**	**7.2%**
**Socioeconomic characteristics**													
**Household wealth quintiles**													
**Q1 (poorest) (ref.)**				-0.759							-0.640		
Q2	0.009	*	0.010	-0.238	-0.002	-4.0		0.006	**	0.005	-0.326	-0.002	-7.1
Q3	0.012	*	0.008	0.156	0.001	2.1		0.012	***	0.009	0.003	0.000	0.1
Q4	0.039	***	0.024	0.341	0.008	13.5		0.007	**	0.006	0.305	0.002	7.0
Q5 (wealthiest)	0.063	***	0.037	0.500	0.018	30.2		0.015	***	0.013	0.658	0.008	33.8
	**Aggregated contribution**				**0.025**	**41.7%**		**Aggregated contribution**				**0.008**	**33.8%**
**Education**													
**No education (ref.)**				-0.101							-0.063		
Pre and Primary	0.023	***	0.071	-0.146	-0.010	-17.0		0.010	**	0.031	-0.328	-0.010	-41.2
Secondary	0.041	***	0.009	0.122	0.001	1.9		0.014	***	0.007	0.154	0.001	4.5
Post-secondary	0.055	***	0.009	0.120	0.001	1.7		0.022	***	0.008	0.237	0.002	8.0
	**Aggregated contribution**				**-0.008**	**-13.5%**		**Aggregated contribution**				**-0.007**	**-28.7%**
**Geographic characteristics**													
**Urban-rural residence**													
**Rural (ref.)**				-0.409							-0.244		
Urban	0.038	***	0.046	0.409	0.019	31.3		0.022	***	0.036	0.244	0.009	36.4
	**Aggregated contribution**				**0.019**	**31.3%**		**Aggregated contribution**				**0.009**	**36.4%**
**Region**													
**BKK (ref.)**				0.029							0.016		
Central	0.062	***	0.058	0.099	0.006	9.4		0.020	***	0.021	0.102	0.002	8.9
North	0.041	***	0.034	-0.075	-0.003	-4.2		0.031	***	0.027	-0.018	0.000	-1.9
Northeast	0.033	***	0.044	-0.241	-0.011	-17.6		0.018	***	0.022	-0.203	-0.004	-18.4
South	0.060	***	0.026	-0.001	0.000	0.0		0.029	***	0.014	-0.027	0.000	-1.5
	**Aggregated contribution**				**-0.008**	**-12.4%**		**Aggregated contribution**				**-0.003**	**-13.0%**
**Health conditions**													
**NCDs morbidity**													
**No morbidity (ref.)**				0.029							0.016		
Single morbidity	-0.107	***	-0.144	-0.018	0.003	4.2		-0.041	***	-0.040	-0.028	0.001	4.6
Multimorbidity	-0.163	***	-0.066	-0.012	0.001	1.3		-0.060	***	-0.046	0.011	-0.001	-2.1
	**Aggregated contribution**				**0.003**	**5.4%**		**Aggregated contribution**				**0.001**	**2.5%**
**Total contribution**					**0.040**	**66.2%**						**0.020**	**80.8%**
**Residuals**					**0.021**	**33.8%**						**0.005**	**19.2%**
**Overall CIs of EQ-5D**					**0.061**	**100.0%**						**0.024**	**100.0%**

**Note**: **Marginal effects (**${\varvec{\beta }}_{\varvec{k}}^{\varvec{m}}$**)** demonstrate associations between determinants and health outcomes (EQ-5D index scores); *** p < 0.001; ** p < 0.01; * p < 0.05; R-squared values of 0.216 (in 2003) and 0.266 (in 2019)

: **Elasticity**$({\varvec{\eta }}_{\varvec{k}}={\varvec{\beta }}_{\varvec{k}}^{\varvec{m}}{\stackrel{-}{\varvec{x}}}_{\varvec{k}})$ is the impact of a determinant on health outcomes with respect to the mean of that determinant; ${\varvec{C}}_{\varvec{k} }$ is Erreygers’ concentration index of each determinant; **Con.** is contributions of each determinant to the overall socioeconomic inequality in health outcomes; **%Con.** is the percentage contribution; **Residual** is unexplained component

It is clearly observed that in 2003 most health outcome inequality (pro-rich inequality) among older adults can be explained by inequalities in household wealth (41.7% contribution), and urban residence (31.3%). Whereas, Thailand’s health security schemes under the UHC policy were not yet a significant contributor to overall inequality due to having no impact on health outcomes.

However, in 2019, Thailand’s health security schemes made the most positive contribution to explaining pro-rich inequality in health outcomes (42.7%). It is the consequence of the fact that the health security scheme (i.e. the UCS) targeting the poorest Thais, who have lower health outcomes when compared to the CSMBS members, as shown by negative elasticity estimation. Additionally, being the UCS members was concentrated among less well-off, as indicated by its negative C_*k*_. Whereas members of CSMBS, and SSS tended to favour the rich. It implied that while the UHC was in place, health outcomes of poor older adults had improved, but not as much as wealthy people. That is probably because the rich older adults have received more benefit from UHC policy than the poor. In other words, there is inequality in health outcomes *within* all three national health security schemes in Thailand.

In 2019, urban residence was the second largest contributor to explaining pro-rich inequality (36.4%)— its contribution increasing from 2003, followed by household wealth (33.8%), whose contribution declined. With respect to age-sex and NCD morbidity, both determinants made only minor contributions to explaining pro-rich inequality.

By contrast, education and region of residence contribute to a pro-poor inequality in 2003 and 2019, as indicated by negative contributions. That is due to both determinants having a high positive impact on health outcomes, despite the highly unequal distribution across the SES spectrum, that would ordinarily have an inequality reducing effect. Also, their contributions found to increase over the study period. This shows that gaps between educational and regional disparities in Thailand have slightly narrowed that would reduce the overall health inequality.

## Discussion

### Decline in socioeconomic inequalities in health outcomes among older adults

The findings state that there exists socioeconomic inequality in health outcomes among older adults (pro-rich inequality), with those in better health more concentrated among the wealthier older adults. However, the degree of inequality *declined* during the period of Thailand’s UHC implementation, and almost reaching parity between the rich and the poor older adults, especially in 2019. Reduced health inequality in this present study may be potentially explained by economic development and improvement in redistribution of income in Thailand since 2000 [32]. Thailand’s Gross Domestic Product (GDP) per capita increased from $3,795 in 2002 to $6,612 in 2019, whereas the Gini coefficient declined from 41.9 to 2002 to 35.0 in 2019. Besides, only 6.2% of Thai population lived below the national poverty line in 2019, a drop from 42.3% to 2000 [50–52]. Regarding the income redistribution, Thailand has implemented various social mechanisms to reduce income inequalities and poverty, such as the UHC and Old-Age pension programs [53], which appeared to protect Thai people, especially older people from poverty and financial catastrophe due to onerous medical payment [32]. As a result of the UHC’s achievement, healthcare coverage increased distinctly, and there has been increased healthcare accessibility and utilization. Particularly, older persons were substantially insured by a free medical care and its coverage increased from 44% to 1991 to 83% in 2001 [54]. Presently, all older people are entitled to receive health benefits from the UHC policy, and they are covered by the UCS (covering 78% of the older population), the CSMBS (20%), and the SSS (2%) [55]. These schemes allow all Thai older persons to achieve good health. Therefore, economic improvement and income redistribution in Thailand could bring about the reduced socioeconomic inequality in health outcomes among older persons in this study.

### Decomposing on socioeconomic inequality in health outcomes: 2003 and 2019

The decomposition analysis of socioeconomic inequality in health outcomes suggests that all three health security schemes under Thailand’s UHC policy were the major contributors in explaining pro-rich inequality in 2019, but not in 2003. This anomaly arises from the fact that Thailand’s UHC was still being rolled out in 2002, and would not have had time to produce a noticeable impact on health outcomes after only a year of implementation. However, its impact was strongly shown in the long run of the UHC in 2019 by improving health outcomes in both the rich and poor older adults and reducing the health outcome gap between them. Moreover, poor older adults seem to have far greater health improvement compared to the rich. There are three possible explanations for the roles of UHC on socioeconomic inequalities in health outcomes among older adults.

First, Thailand’s UHC had been implemented to improve the health for all and reduce health inequalities, particularly the poorest population, as it enhances access to care without financial hardship [56]. Older people are one of the target groups in the pro-poor policy (i.e., the UCS) and have enjoyed health benefits to improve their health outcomes [12]. Second, Thailand has a strong primary health care (PHC) system that sustained the performance of the overall system over the past 50 years and has enhanced the UHC’s achievements [27, 57]. The older lower-income in rural areas have more access to and more frequent use of healthcare services at PHC facilities close to their homes and receive any necessary referrals [17, 25, 29]. Third, the UCS itself makes ongoing efforts to expand the benefits package and services tailored to the evolving Thai population’s needs and boosted financial protection. Beneficiaries enjoy additional high-cost treatments (e.g., anti-retroviral treatment, renal replacement therapy, chemotherapy, radiation therapy, stem-cell transplant) and medication benefits. Moreover, the UCS included new interventions for health promotion and disease prevention (e.g., diabetes and hypertension screening, health literacy, etc.); rehabilitation interventions for older people (e.g. the Long-Term Care program); and emergency medical care [21, 25]. Currently, health promotion and disease prevention, and rehabilitation services are available for all Thai citizens, regardless whether they are CSMBS and SSS members [28].

In these three senses, Thailand’s UHC has consistently played a crucial role in enhancing health outcomes. However, as indicated in this study, there is inequality in health outcomes within all three public health security schemes in Thailand, as the UCS was concentrated among the less well-off older adults, and the UCS members were found to have lower health outcomes than the CSMBS members. That could be a result of the different details between each scheme in terms of the benefit package design, service access, capital expenditure, and finance source, particularly payment mechanisms differentials between the UCS and CSMBS. Despite the fact that both CSMBS and UCS are solely financed by general tax revenues, the payment methods to service provider are literally distinct. The UCS has applied a completely close-ended provider payment (i.e., fixed capitation) for outpatient care, and the diagnosis-related groups (DRG) under a global budget for purchasing inpatient care. The UCS payment appears to be a strong cost-containment and financial sustainability [24, 58]. However, under the close-ended payment, UCS beneficiaries can access mainly medicines included in the National List of Essential Medicines (NLEM). This might reflect the under-utilization in UCS services [59]. Compared with CSMBS, which used fee-for-service method claims by hospitals for outpatient care, and DRG without a global budget ceiling for inpatient care [58]. CSBMS beneficiaries were found to enjoy more on a variety of comprehensive services and have more choices of medicines beyond the NLEM lists, especially drugs for controlling NCDs, as well as have longer period to stay in the hospital [24, 25, 60]. The differences in payment mechanisms between the UCS and CSMBS may result in disparities in individual access to healthcare and health outcomes [23]. Therefore, our findings suggest that it is important to minimize differences across schemes, especially the UCS and CSMBS, as this might improve Thai older people’s health outcomes and narrow health inequalities while also maximizing healthcare efficiency.

Our study also reported that urban residence was the second largest contributor to explaining pro-rich inequality in 2019, its contribution increasing from 2003. This study highlights that the gap in health inequalities among older adults in urban and rural areas has not been significantly reduced and has to be solved. Even though Thailand had a huge rural health development and achieved full geographical coverage of health delivery systems prior to UHC in 2002 [21, 32], there are still significant gaps between older people in urban and rural areas. Older people in rural and remote areas still face more challenges due to travel distance, lack of public or private and affordable transportation, and poor roads that hinder access to health facilities and higher-level facilities [17, 30]. Additionally, an insufficient number of health professionals practicing in rural areas (based on the population) remains a significant and ongoing vital problem [61]. Hence, closing rural-urban gaps in healthcare accessibility and health professional distribution in rural areas would reduce health inequalities among older adults in Thailand.

A common theme in this study is that household wealth made a positive contribution in explaining pro-rich inequality, but its contribution declined over the study period. This observation is probably due to the redution in unequal distribution of household wealth. The proportion of older people (age 60 or older) who were living under the poverty line decreased from 46.5% to 2002 to 34.3% in 2015 [62]. In addition, since 2009, older people were provided greater economic security, in Thailand, by receiving the monthly Old-Age pension (600-1,000 baht or approximately 18–30 US$) [63]. Another possible explanation is Thailand’s UHC achievement, which has led to a low incidence of catastrophic health spending in both the richest and poorest households [32]. The evidence in 2011 shows that the incidence of catastrophic expenditures among Thai older persons in the poorest and poor households (Q1 and Q2) were about 1% and 2%, respectively [17]. This low incidence took place in the midst of a significant increase in healthcare utilization, especially among poor people or older persons who are the UCS members [64, 65]. Therefore, older individuals living in low- socioeconomic households would be shielded from poverty and direct healthcare spending.

### Limitations

Some limitations of the study are worth mentioning. First, there may possibly introduce recall bias in self-reported information regarding EQ-5D-5L questionnaire in HWS years 2003 and 2006. This is because the EQ-5D-5L questionnaire in 2003 and 2006 asked respondents to describe the best health outcomes within the past “one month.” Whereas, the HWS in 2015 and 2019 asked to choose best describes their health in “today.” Second, the HWS did not provide information about being in the previous health security scheme. Generally, the SSS beneficiaries are transferred to the UCS after retirement. Their health status might be made by the health benefits from the SSS throughout their working life. Lastly, this study was limited by the cross-sectional design. Thus, longitudinal studies are recommended to assess the trend of socioeconomic inequality in health outcomes of older adults and decompose the true causes of socioeconomic inequality in health.

## Conclusion

Thailand’s UHC achievement has reduced the barriers to universal access to basic healthcare services, eventually improving population health. However, challenges in health inequalities among the older population remain. This study provides empirical evidence of a persistence effect of socioeconomic inequality on health outcomes among older adults; better health outcomes are concentrated among the rich older adults (pro-rich inequality), but appears to decline during the UHC implementation. It seems that the rich and poor older adults will soon have equal opportunity to be healthy. To further understand and identify the potential effect of UHC and other health determinants on health inequality, this study demonstrates that Thailand’s health security schemes, urban residence, and household wealth are the major positive contributors to explaining the pro-rich inequality among older Thai adults. This study highlights that there are health inequalities within all three health security schemes in Thailand that need to be addressed. Therefore, this study advocates for government to minimize differences among schemes for better equity, efficiency and fairness, for instance, by delivering healthcare services with a consistent standard across all schemes, harmonizing benefits package multiple schemes, and narrowing the gap’s capital expenditures between the UCS and CSMBS. This could make it possible for older people in the UCS to have comparable or even better health outcomes than those in the CSMBS. Moreover, it is necessary to make Thailand’s UHC sustainable in the long run to deal with the consequences of rapidly aging populations, as demand and expenditure for all healthcare services increase.

## Data Availability

All datasets used in this study are available from the NSO with some restrictions. The data are not publicly available. For using the data, it needs to be approved by the NSO.

## List of abbreviations

UHC: Universal Health Coverage
UCS: Universal Coverage Scheme
CSMBS: Civil Servant Medical Benefit Scheme
SSS: Social Security Scheme
EQ-5D: EuroQol 5 dimensions

## Footnotes

1 The largest sample size in 2015 was due to the sampling frame of NSO covered all 13 regions of the National Health Security Office (NHSO region), whereas the sampling frames in other survey years covered five geographic regions in Thailand.

2 Cronbach’s alpha = 0.867 (in 2003), 0.841 (in 2006), 0.857 (in 2015), 0.844 (in 2019).

3 Reporting unknown were 3.0% in 2003, 2.3% in 2006, 0.5% in 2015, and 0.2% in 2019; reporting private health insurance were 1.1% in 2003, 1.0% in 2006, 0.7% in 2015, and 0.5% in 2019.

4 In the HWS data sets, the EQ-5D index had a ceiling effect with about 32% (in 2003), 34% (in 2006), 54% (in 2015), and 56% (in 2019) of older adults reporting full health.
